# Supplementary material for: tACS motor system effects can be caused by transcutaneous stimulation of peripheral nerves
Source: Nat Commun. 2019 Jan 17;10:266. doi: 10.1038/s41467-018-08183-w (PMC6336776; doi:10.1038/s41467-018-08183-w)
Supplement: Supplementary file 1 — Supplementary Information [file 41467_2018_8183_MOESM1_ESM.pdf]

Supplementary Information

tACS motor system effects can be caused by transcutaneous stimulation of peripheral nerves

Asamoah et al.

Supplementary Results

Experiment 1

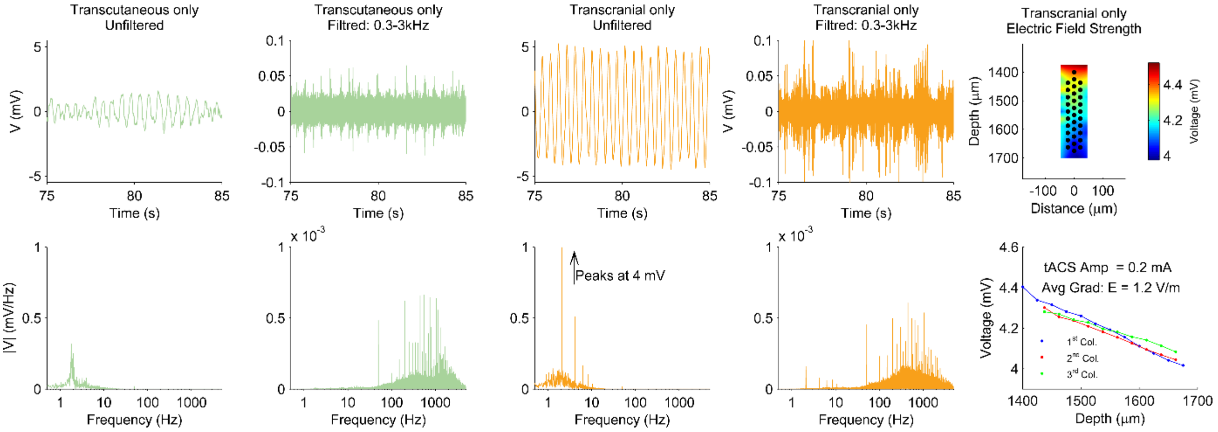

Supplementary Figure 1. Voltage waveforms recorded during transcutaneous-only and transcranial-only stimulation and the calculation of the electric field strength.

Columns 1 and 3 show the time-series (top) and Fourier transform (bottom) of the unfiltered recordings during transcutaneous-only (green) and transcranial-only (orange) stimulation respectively. The voltage signal during transcranial-only stimulation is large because of volume conduction of the stimulation signal from the transcranial stimulating screws to the electrode recording site in the brain. Note the large peak (4 mV, off scale) in the Fourier transform at the stimulation frequency. During transcutaneous-only stimulation, there is little, if any, volume conduction of the stimulation signal from the stimulating electrodes on the rat's body to the recording electrodes in the brain. The broad peak in the Fourier transform during transcutaneous stimulation, centered around 2 Hz, is due to the endogenous brain oscillation. The stimulation frequency was always chosen to approximately match the frequency of this endogenous brain oscillation.

Columns 2 and 4 show the same information as columns 1 and 3 but band-passed at 0.3-3 kHz to extract the spiking activity. The electrical artifact at the stimulation frequency (2Hz) in the transcranial-only condition has been completely removed by the filtering.

The bottom panel on the far right column shows an example of the voltage amplitude of Fourier component at the stimulation frequency during transcranial-only stimulation for each of the 32 electrodes (extracted from the unfiltered data). Electrodes from each of the three electrode-columns are joined by a line and shown as different colors (blue, red, green). The electric field strength in the dorso-ventral direction (i.e. the electrode penetration direction) was calculated as the spatial gradient of the voltage component at the stimulation frequency. The upper panel on the far left column shows an estimate of this electric field strength at each electrode site (black dots). Averaging across all animals and all stimulation sites shows that a transcranial-only stimulation amplitude of 0.1 mA (peak-amplitude) generated an electric field strength of 0.9 V/m. The relationship was linear meaning that on average an amplitude of 0.2 mA generated an electric field strength of 1.8 V/m.

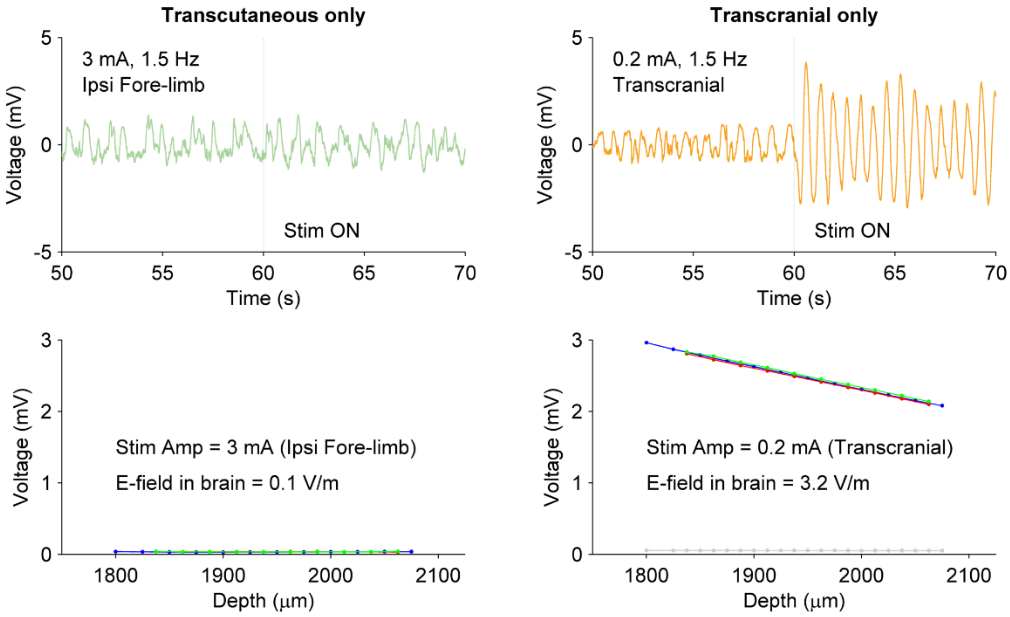

Supplementary Figure 2. Comparison of voltage waveforms recorded during transcutaneous-only and transcranial-only stimulation and the calculation of the electric field strength.

Since the amplitudes used in the transcutaneous-only conditions were higher than in the transcranial-only condition there was a possibility that some of the current from the limb reached the brain through volume conduction. If this volume conducted electric field were strong enough, it could be that the neural entrainment observed during the transcutaneous-only condition was caused by direct modulation of the cortical neurons (similar to the transcranial-only condition) and not indirectly by stimulation of peripheral nerves in the limb. However, we found this not to be the case. The top panels above show an example of how the signal recorded on one channel from the 32-channel probe changed when transcutaneous-only (left) and transcranial-only (right) stimulation was switched on. As expected transcranial-only stimulation created a large amplitude electrical signal in the brain, while transcutaneous-only (ispi fore-limb in this example) stimulation did not create a noticeable electric field in the brain. The bottom two panels show how the electric field strength was calculated for each stimulation condition (same as in the figure above, see Methods for details). The colored lines (blue, red and green) show the voltage on each electrode column during stimulation and the grey lines at the bottom show the voltage on each electrode before stimulation. There are large increases in voltage for transcranial-only stimulation but not for transcutaneous-only stimulation (colored lines overlap the grey lines).

We calculated the electric field strength in the brain for all four transcutaneous-only conditions at all recording sites and in all animals. We found only very weak electric fields in the brain during limb stimulation. Values averaged across all recordings for 3 mA transcutaneous-only were: contra-hind 0.075 V/m; contra-fore 0.067 V/m; ipsi-hind 0.017 V/m; ipis-fore 0.041 V/m. These values are actually in the same range as the electric field strength of the local field potential during the stimulation off conditions: 0.037 V/m averaged during all transcutaneous-only conditions and 0.041 V/m for all transcranial-only conditions. Thus, the small electric field measured during transcutaneous-only stimulation is likely due to the local field potential and not volume conduction of current from the stimulated limb. Even if it were due to volume conduction, it is still well below the 0.9 V/m needed to cause direct neural entrainment of cortical neurons.

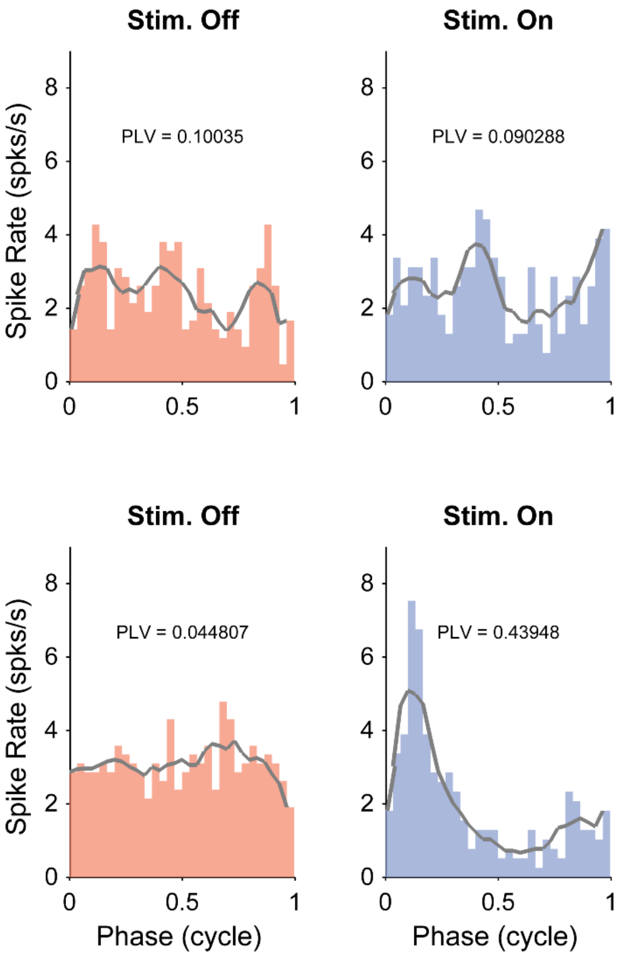

Supplementary Figure 3. An example highlighting some of the variability in response to transcutaneous-only stimulation. At the group level, we observed an increase in neural entrainment for all four transcutaneous-only configurations tested (Contra-fore, Ipsi-fore, Contra-hind and Ipsi-hind). However, there was a large amount of variability between different neurons. This is summarized in Supplementary Table 3 (below). This figure shows an example of one neuron that did not entrain to contralateral hind-limb stimulation (top row, no increase in PLV from OFF to ON conditions) but did show strong entrainment to contralateral fore-limb stimulation (bottom row, large increase in PLV from OFF to ON).

It is worth noting that not all neurons showed entrainment. In general about 75 % of neurons showed an increase in PLV from the OFF to the ON condition for both transcranial and transcutaneous stimulation. The other 25% of neurons either showed no change in PLV or decreased in PLV. All neurons were included in the group analysis present above. As one would expect, the percentage of neurons showing entrainment generally increased as the stimulation amplitude was increased for all stimulation configurations (full results are presented in Supplementary Results, Table 3). It should also be noted that the amount of neural entrainment measured in the low amplitude conditions (e.g. Fig. 3 top row) is not large and it is unclear what, if any, effect this level of entrainment would have on behavior. Higher amplitude stimulation did cause stronger neural entrainment (e.g. Fig. 3 bottom row) which may be expected to influence behavior. In line with this, we have previously shown that electric field strengths of around 8 V/m are need to cause a measurable effect on limb movements induced in an anesthetized rat (see Figure 2 in Khatoun et al, J. Neurosci, 2017).

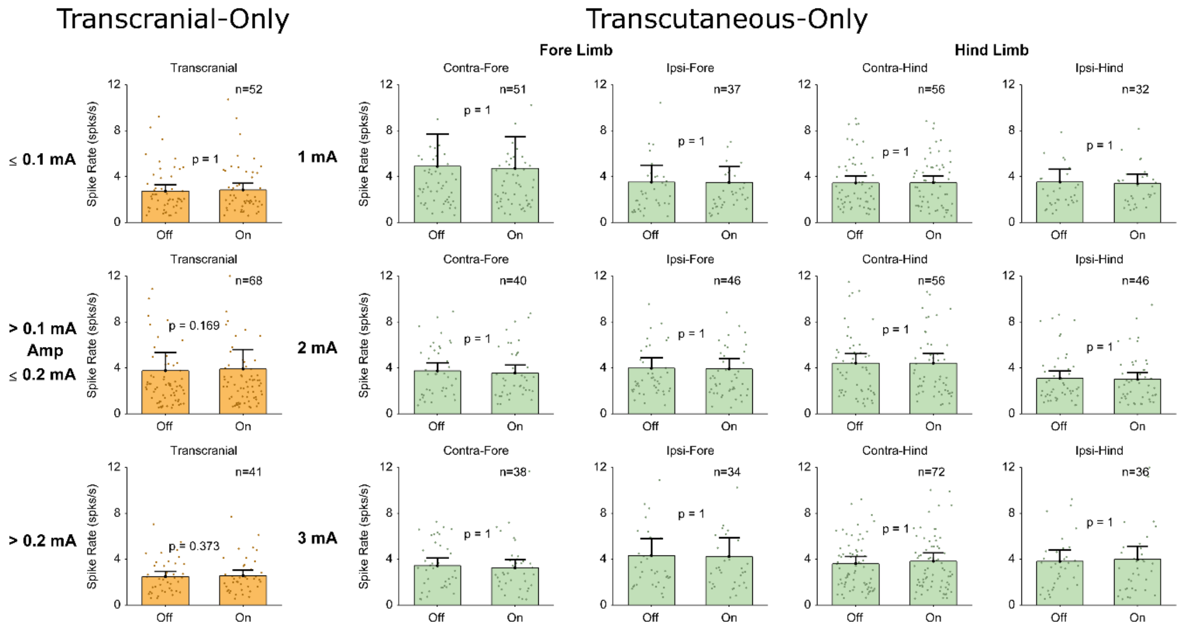

Supplementary Figure 4. Group bar charts showing the effect of transcranial-only (orange) and transcutaneous-only (green) stimulation on spike-rate. The rows show the effect of increasing stimulation amplitude. The transcranial-only data is grouped into three amplitude ranges: LOW (≤0.1 mA), MEDIUM (>0.1 mA, but ≤0.2 mA), HIGH (>0.2 mA). While the transcutaneous-only data was collected at 3 fixed amplitudes: LOW (1 mA), MEDIUM (2 mA), HIGH (3 mA). The bar graphs compare the mean spike-rate for stimulation OFF and ON for each amplitude condition. Dots show individual data points. Error bars show the confidence intervals. For transcutaneous-only stimulation four different electrode configurations were tested: contralateral (to the 32-channel recording probe) fore, contralateral hind, ipsilateral fore and ipsilateral hind. There was never a significant increase in spike-rate from stimulation OFF to ON for any of the conditions tested (Wilcoxon signed rank test, one-tailed, Bonferroni corrected p-vales are reported – transcranial n=3, transcutaneous n=12). Thus, we found neither transcranial-only nor transcutaneous-only stimulation caused a significant increase in spike-rate for any of the stimulation amplitudes tested. Together with the data shown in Fig 3 in the main manuscript, this indicates that both transcranial-only and transcutaneous-only stimulation can affect spike-timing to cause neural entrainment without actually increasing spike-rate at the group level.

Supplementary Table 1. A complete list of all the single-neurons (after spike sorting) and stimulation parameters from which transcranial-only stimulation data was collected. The columns list the rat number (Rat), date of the experiment (Date), penetration (Pen), depth (Depth, µm), neuron number (Neuron), the frequency at which transcranial-only stimulation was applied (Freq, Hz) and the different amplitudes (Amplitudes, mA).

**Rat**	**Date**	**Pen**	**Depth**	**Neuron**	**Freq**	**Amplitudes**
R14	17/11/2017	2	1400	1	1.6	0.025 0.05 0.1 0.2
				2	1.6	0.025 0.05 0.1 0.2
				3	1.6	0.05
				4	1.6	0.05
	20/12/2017	3	850	5	2	0.1
				6	2	0.1
				7	2	0.1
R15	8/12/2017	1	1100	8	1.5	0.1
				9	1.5	0.1
				10	1.5	0.1
				11	1.5	0.1
			1400	12	2.1	0.2
				13	2.1	0.2
			1800	14	1.5	0.2
				15	1.5	0.2
				16	1.5	0.2
				17	1.5	0.2
				18	1.5	0.2
				19	1.5	0.2
	24/11/2017	1	1250	20	2	0.05
R16	13/12/2017	2	2030	21	2	0.15
	18/12/2017	1	2300	22	2	0.12
				23	2	0.12
				24	2	0.12
R17	1/12/2017	1	1200	25	1.4	0.1 0.2
				26	1.4	0.2
			1350	27	2	0.12
			2100	28	2	0.2 0.5
				29	2	0.2 0.5
				30	2	0.2 0.5
				31	2	0.2 0.5
				32	2	0.2
				33	2	0.2
				34	2	0.2
				35	2	0.2
				36	2	0.2
	15/12/2017	1	1100	37	1	0.1
			1800	38	1.5	0.2
				39	1.5	0.2
				40	1.5	0.2

				41	1.5	0.2
				42	1.5	0.2
				43	1.5	0.2
				44	1.5	0.2
R18	6/12/2017	1	1450	45	1.6	0.025 0.1
			1650	46	1.8	0.25
				47	1.8	0.25
				48	1.8	0.25
R19	16/03/2018	1	950	49	1.5	0.05 0.15 0.25
				50	1.5	0.05 0.15 0.25
				51	1.5	0.05 0.15 0.25
				52	1.5	0.05 0.15 0.25
				53	1.5	0.05 0.15 0.25
				54	1.5	0.05 0.15 0.25
				55	1.5	0.05 0.25
				56	1.5	0.05
			951	57	1.5	0.05 0.15 0.25
				58	1.5	0.05 0.15 0.25
				59	1.5	0.05 0.15 0.25
				60	1.5	0.05 0.15 0.25
				61	1.5	0.05 0.15
				62	1.5	0.05 0.15
				63	1.5	0.15
				64	1.5	0.15
				65	1.5	0.15
				66	1.5	0.15
			1400	67	1.5	0.25
				68	1.5	0.25
				69	1.5	0.25
			2000	70	1.5	0.05 0.15 0.25
				71	1.5	0.05
		3	1000	72	1.5	0.05 0.15
			1300	73	1.5	0.05 0.15 0.25
				74	1.5	0.05 0.15 0.25
				75	1.5	0.05 0.15 0.25
				76	1.5	0.15 0.25
			2300	77	1.5	0.05 0.15 0.25
				78	1.5	0.15 0.25
				79	1.5	0.15
				80	1.5	0.15
R20	22/03/2018	1	1200	81	1.5	0.3
			1500	82	1.5	0.1 0.2 0.3
				83	1.5	0.3
			1850	84	1.5	0.1 0.2 0.3
				85	1.5	0.1 0.3
				86	1.5	0.1 0.3

			2150	87	1.5	0.1 0.2 0.3
				88	1.5	0.1 0.2 0.3
				89	1.5	0.1 0.3
		2	1100	90	1	0.2
			1450	91	1.5	0.1 0.2 0.3
				92	1.5	0.1
				93	1.5	0.1
			1790	94	1.5	0.08 0.18 0.28
				95	1.5	0.18 0.28
				96	1.5	0.18
			2070	97	1.5	0.4

Supplementary Table 2. A complete list of all the single-neurons (after spike sorting) and stimulation parameters from which transcutaneous-only stimulation data were collected. The columns list the rat number (Rat), date of the experiment (Date), penetration number (Pen), depth (Depth, μm), neuron number (Neu), the electrode configurations tested (contra-hind, CHind; contra-fore, CFore; ipsi-hind; IHind; ipsi-fore, IFore), the frequency at which transcutaneous-only stimulation was applied (Freq, Hz) and the different amplitudes (Amps, mA). Note, the neuron numbering does not correspond to the numbering in Supplementary Table 1. The last row shows the total number of neurons for each of the four stimulation configurations.

**Rat**	**Date**	**Pen**	**Depth**	**Neu**	**CHind**	**CFore**	**IHind**	**IFore**	**Freq**	**Amps**
R14	20/12/2017	3	850	1	Yes	Yes	Yes	Yes	2	1 3
				2	Yes	Yes	No	Yes	2	1
			1550	3	Yes	Yes	Yes	No	1	1 2 3
				4	Yes	Yes	Yes	No	1	1 2 3
				5	Yes	Yes	Yes	No	1	1 2 3
				6	Yes	Yes	Yes	No	1	1 2 3
				7	Yes	Yes	Yes	No	1	1 2 3
				8	Yes	Yes	Yes	No	1	1 2 3
				9	Yes	No	Yes	No	1	2 3
				10	Yes	No	Yes	No	1	3
				11	Yes	No	Yes	No	1	3
				12	Yes	No	Yes	No	1	3
				13	Yes	No	No	No	1	3
				14	Yes	No	No	No	1	3
				15	Yes	No	No	No	1	3
R15	8/12/2017	1	1400	16	Yes	No	No	No	2	1 2
				17	Yes	No	No	No	2	1
				18	Yes	No	No	No	2	1
				19	Yes	No	No	No	2	1
				20	Yes	No	No	No	2	1
			1800	21	Yes	No	No	No	1.5	2
				22	Yes	No	No	No	1.5	2
				23	Yes	No	No	No	1.5	2
				24	Yes	No	No	No	1.5	2
				25	Yes	No	No	No	1.5	2
				26	Yes	No	No	No	1.5	2
				27	Yes	No	No	No	1.5	2
				28	Yes	No	No	No	1.5	2
R16	13/12/2017	2	2030	29	Yes	No	No	No	2	1 2 3
				30	Yes	No	No	No	2	1 2 3
				31	Yes	No	No	No	2	1
		3	1500	32	Yes	Yes	Yes	No	2.5	3
				33	Yes	No	No	No	2.5	3
				34	Yes	No	No	No	2.5	3
				35	Yes	No	No	No	2.5	3

				36	Yes	No	No	No	2.5	3
				37	Yes	No	No	No	2.5	3
				38	Yes	No	No	No	2.5	3
				39	Yes	No	No	No	2.5	3
			1750	40	Yes	Yes	Yes	Yes	1	1 2 3
				41	Yes	Yes	Yes	Yes	1	1 2 3
				42	Yes	Yes	Yes	Yes	1	1 2 3
				43	Yes	Yes	Yes	Yes	1	1 2 3
				44	Yes	Yes	No	Yes	1	1 2 3
				45	Yes	Yes	No	Yes	1	1 2 3
				46	Yes	Yes	No	No	1	1 2 3
	18/12/2017	1	1980	47	Yes	Yes	Yes	Yes	2	3
			2300	48	Yes	Yes	Yes	Yes	2	1 2 3
				49	Yes	Yes	Yes	Yes	2	1 2 3
				50	Yes	Yes	Yes	Yes	2	1 3
				51	Yes	No	Yes	Yes	2	1 3
				52	Yes	No	Yes	Yes	2	1
R17	15/12/2017	1	1100	53	Yes	No	Yes	Yes	1	3
				54	Yes	No	Yes	Yes	1	3
			1800	55	Yes	Yes	Yes	Yes	1.5	1 2 3
				56	Yes	Yes	Yes	Yes	1.5	1 2 3
				57	Yes	Yes	Yes	Yes	1.5	1 2 3
				58	Yes	Yes	Yes	Yes	1.5	1 2 3
				59	Yes	Yes	Yes	Yes	1.5	1 2 3
				60	Yes	Yes	Yes	Yes	1.5	1
				61	Yes	Yes	Yes	Yes	1.5	1
R18	6/12/2017	1	1450	62	Yes	No	No	No	1.6	1
				63	Yes	No	No	No	1.6	1
				64	Yes	No	No	No	1.6	1
				65	Yes	No	No	No	1.6	1
				66	Yes	No	No	No	1.6	1
				67	Yes	No	No	No	1.6	1
				68	Yes	No	No	No	1.6	1
			1650	69	Yes	No	No	No	1.8	2 3
				70	Yes	No	No	No	1.8	2 3
				71	Yes	No	No	No	1.8	2 3
				72	Yes	No	No	No	1.8	3
				73	Yes	No	No	No	1.8	3
				74	Yes	No	No	No	1.8	3
R19	16/03/2018	1	950	75	Yes	Yes	Yes	Yes	1.5	1 2 3
				76	Yes	Yes	Yes	Yes	1.5	1 2 3
				77	Yes	Yes	Yes	Yes	1.5	1 2 3
				78	Yes	Yes	Yes	No	1.5	1 2 3

				79	Yes	No	Yes	No	1.5	1 2 3
				80	Yes	No	Yes	No	1.5	1 2 3
				81	Yes	No	No	No	1.5	2 3
		3	1300	82	Yes	Yes	Yes	Yes	1.5	1 2 3
				83	Yes	Yes	No	Yes	1.5	1 2 3
				84	Yes	No	No	Yes	1.5	1 3
				85	Yes	No	No	Yes	1.5	1 3
R20	22/03/2018	1	1200	86	Yes	No	No	No	1.5	3
			1500	87	Yes	Yes	Yes	Yes	1.5	2
				88	Yes	Yes	Yes	No	1.5	2
				89	Yes	Yes	No	No	1.5	2
			1850	90	Yes	Yes	Yes	Yes	1.5	1 2 3
				91	Yes	Yes	Yes	Yes	1.5	1 2 3
				92	Yes	Yes	Yes	Yes	1.5	2 3
				93	Yes	Yes	Yes	Yes	1.5	2 3
				94	Yes	Yes	Yes	Yes	1.5	2 3
				95	Yes	No	No	Yes	1.5	3
				96	Yes	No	No	Yes	1.5	3
				97	Yes	No	No	Yes	1.5	3
			2150	98	Yes	Yes	Yes	Yes	1.5	1 2 3
				99	Yes	Yes	Yes	Yes	1.5	1 2 3
				100	Yes	Yes	Yes	Yes	1.5	1 2
				101	Yes	Yes	Yes	Yes	1.5	1
				**Total**	101	45	49	43		

Supplementary Table 3. A list of the percentage of neurons showing either an increase in neural entrainment from the OFF to the ON condition (% Neurons with PLV increase) and the percentage showing either no change or a decrease in neural entrainment from the OFF to the ON condition (% Neurons with PLV decrease) for each stimulation configuration and amplitude.

**Configuration (Amplitude)**	**% Neurons with PLV increase**	**% Neurons with PLV decrease**
**Transcranial (All)**	83	17
Transcranial (<0.1 mA)	77	23
Transcranial (>0.1 mA <0.2 mA)	82	18
Transcranial (>0.2 mA)	93	7
**Contra-fore (All)**	74	26
Contra-fore (1 mA)	67	33
Contra-fore (2 mA)	68	33
Contra-fore (3 mA)	89	11
**Ipsi-fore (All)**	79	21
Ipsi-fore (1 mA)	73	27
Ipsi-fore 2 mA)	80	20
Ipsi-fore (3 mA)	82	18
**Contra-hind (All)**	67	33
Contra-hind (1 mA)	59	41
Contra-hind (2 mA)	70	30
Contra-hind (3 mA)	71	29
**Ipsi-hind (All)**	71	29
Ipsi-hind (1 mA)	78	22
Ipsi-hind 2 mA)	65	35
Ipsi-hind (3 mA)	72	28

Experiment 2A

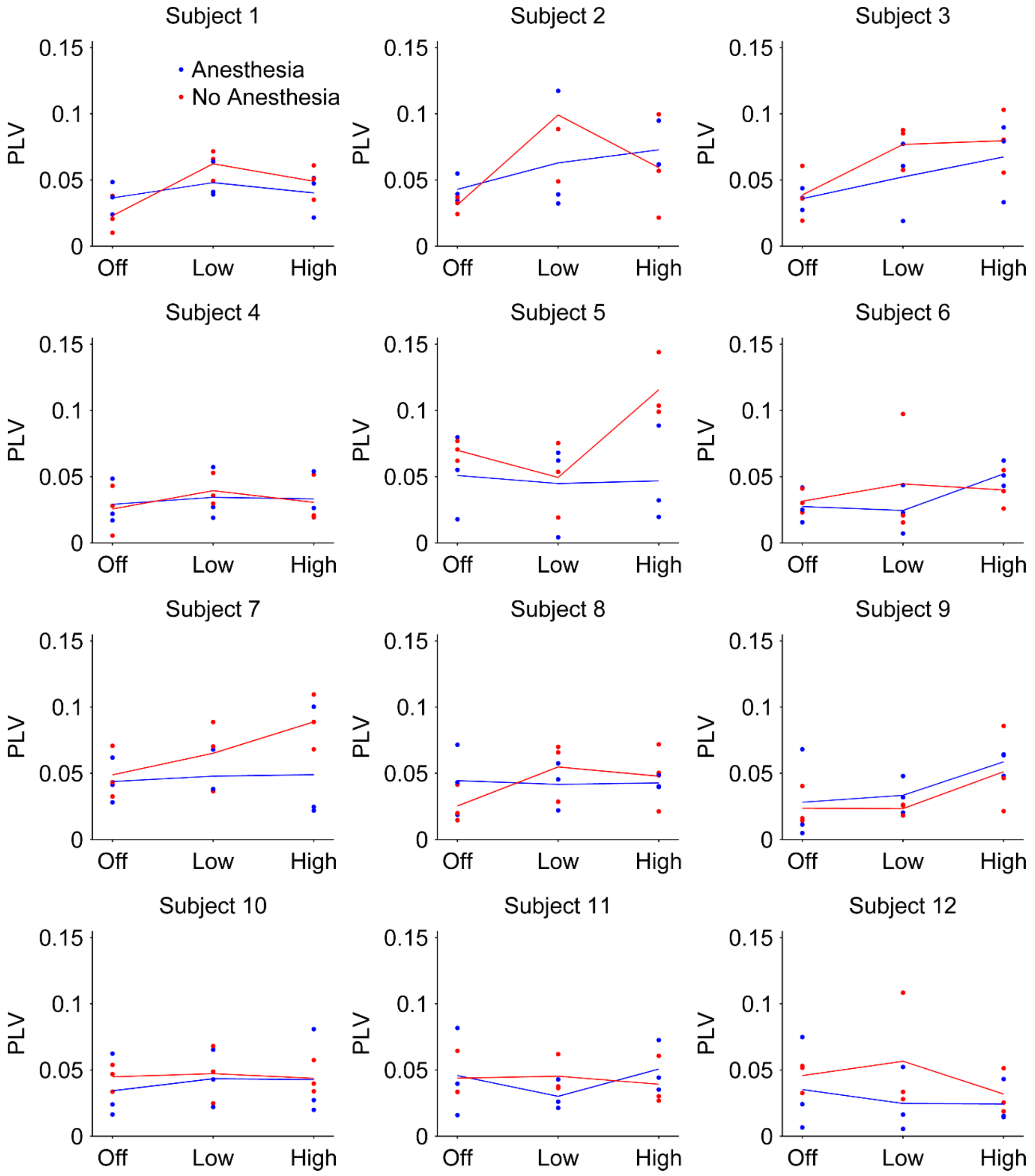

Supplementary Figure 5. Individual subject data showing tACS entrainment of physiological tremor in healthy volunteers: effects of amplitude and topical anesthesia (Experiment 2A). Each panel shows data from one subject. Data from the no anesthesia condition are shown in red and data from the anesthesia condition are shown in blue. Single dots represent the PLV value for one of the three repetitions collected for each condition. The lines show the mean PLV for each condition. Similarly to other tACS studies, there is considerable response variability. Some subjects show a large increase in PLV with increasing stimulation amplitude in the no anesthesia condition (e.g. Subjects 3, 5 and 7), while others show very little effect (e.g. Subjects 8, 11 and 12). For most subjects who showed an increase in PLV in the no anesthesia condition, we found that PLV decreased under the anesthesia condition. This indicates that a transcutaneous mechanism, and not a transcranial mechanism, was causing tremor entrainment. A linear mixed model was applied to the group level data and confirmed these observations (see Results in main text). Although our samples are not normally distributed, some readers may be more familiar with parametric statistics. A two-way repeated measures ANOVA showed similar results (amplitude effect $F(2,22)=5.963$, $p = 0.008$, anesthesia effect $F(1,11) = 5.804$, $p = 0.035$, interaction $F(2,22) = 2.555$, $p = 0.100$) and lead to the same conclusions.

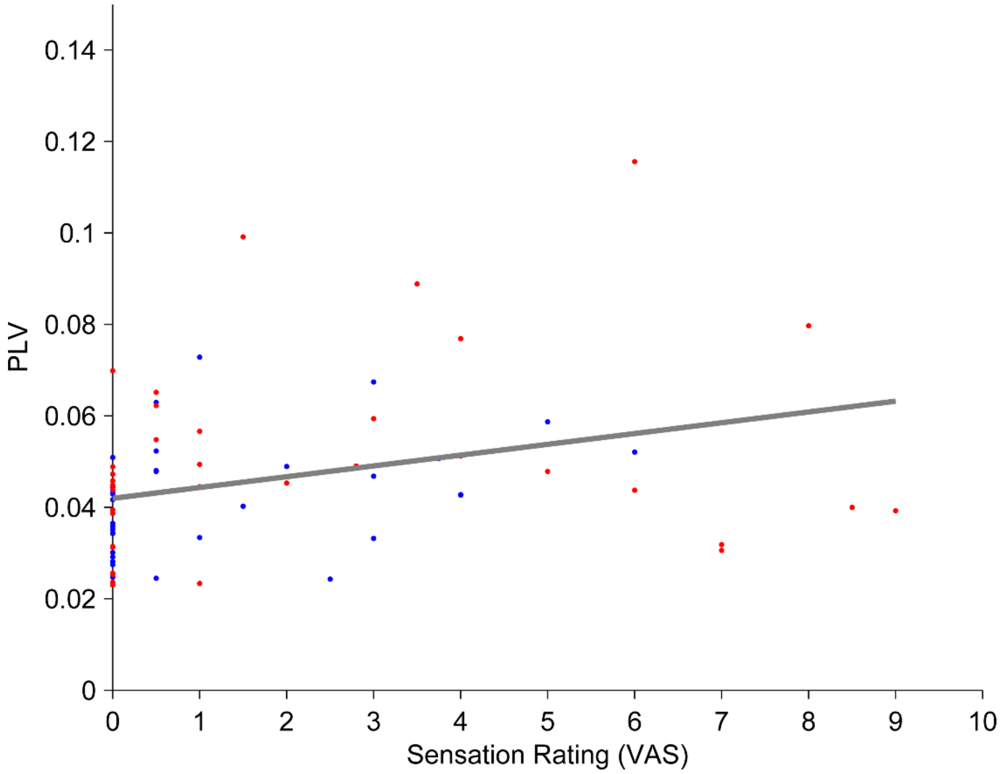

Supplementary Figure 6. The relationship between sensation intensity rating and tremor entrainment (Experiment 2). The dots show the average PLV value for each anesthesia condition (blue – anesthesia; red – no anesthesia) and amplitude condition (not differentiated) for all subjects, plotted as a function of the corresponding sensation intensity rating for that particular condition. The grey line shows the fit from a linear mixed model where PLV was estimated using sensation rating as a fixed effect and subject as a random effect (MATLAB, fitlme.m, model notation: PLV ~ 1 + Sensation Rating + (1 | Subject)). We found a significant effect of sensation rating on tremor entrainment (p=0.001201). This means that subjects who rated the sensation of the stimulation as being more intense, in general showed higher levels of tremor entrainment. If tACS effects are caused by transcutaneous stimulation of peripheral nerves in the skin, then we would expect to see this type relationship between tremor entrainment and sensation rating.

Supplementary Table 4. The amplitudes used to test each of the 12 subjects in Experiment 2 for the anesthesia conditions and the low and high amplitude conditions. The last row shows the mean amplitude for each condition tested. An estimate of the electric field strength in the scalp and brain, calculated from the electro-anatomical model, is also provided for each condition.

**Subject**	**Anesthesia**		**No Anesthesia**		**Anesthesia**		**No Anesthesia**	
	**Low (mA)**	**High (mA)**	**Low (mA)**	**High (mA)**	**Low E (V/m) (Skin/Brain)**	**High E (V/m) (Skin/Brain)**	**Low E (V/m) (Skin/Brain)**	**High E (V/m) (Skin/Brain)**
1	1	2	1	2	23.5/0.13	47/0.26	23.5/0.13	47/0.26
2	1	2	1	2	23.5/0.13	47/0.26	23.5/0.13	47/0.26
3	1	2	1	2	23.5/0.13	47/0.26	23.5/0.13	47/0.26
4	0.16	0.8	0.16	0.8	3.76/0.02	18.80/0.10	3.76/0.02	18.80/0.10
5	0.5	2.5	0.5	2.5	11.75/0.07	58.75/0.33	11.75/0.07	58.75/0.33
6	0.5	2.5	0.5	2.5	11.75/0.07	58.75/0.33	11.75/0.07	58.75/0.33
7	0.5	2.5	0.3	1.5	11.75/0.07	58.75/0.33	7.05/0.04	35.25/0.2
8	0.5	2.5	0.5	2.5	11.75/0.07	58.75/0.33	11.75/0.07	58.75/0.33
9	0.5	2.5	0.5	2.5	11.75/0.07	58.75/0.33	11.75/0.07	58.75/0.33
10	0.5	2.5	0.5	2.5	11.75/0.07	58.75/0.33	11.75/0.07	58.75/0.33
11	0.5	2.5	0.5	2.5	11.75/0.07	58.75/0.33	11.75/0.07	58.75/0.33
12	0.5	2.5	0.5	2.5	11.75/0.07	58.75/0.33	11.75/0.07	58.75/0.33
**Mean**	**0.597**	**2.233**	**0.58**	**2.15**	**14.03/0.08**	**52.48/0.29**	**13.63/0.08**	**50.53/0.28**

Experiment 2B

Table 5. Essential tremor patient details. Six patients participate in the no anesthesia experiment (No) and six in the anesthesia experiment (Yes). An estimate of the electric field strength in the scalp and brain, calculated from the electro-anatomical model, is also provided for each patient.

Patient number	Age	Tremor Frequency (Hz)	Anesthesia	tACS (mA)	Brain E-Field (V/m)	Skin E-Field (V/m)
1	83	2.5	No	2	0.26	47
2	56	3.5	No	2	0.26	47
3	79	3	No	2	0.26	47
4	75	3.5	No	2	0.26	47
5	68	4	No	2	0.26	47
6	70	3.5	No	2	0.26	47
7	67	4.5	Yes	3.5	0.45	82.25
8	76	3.5	Yes	5	0.65	117.5
9	76	3	Yes	5	0.65	117.5
10	72	4	Yes	5	0.65	117.5
11	55	4	Yes	5	0.65	117.5
12	77	3.5	Yes	4.5	0.58	105.75

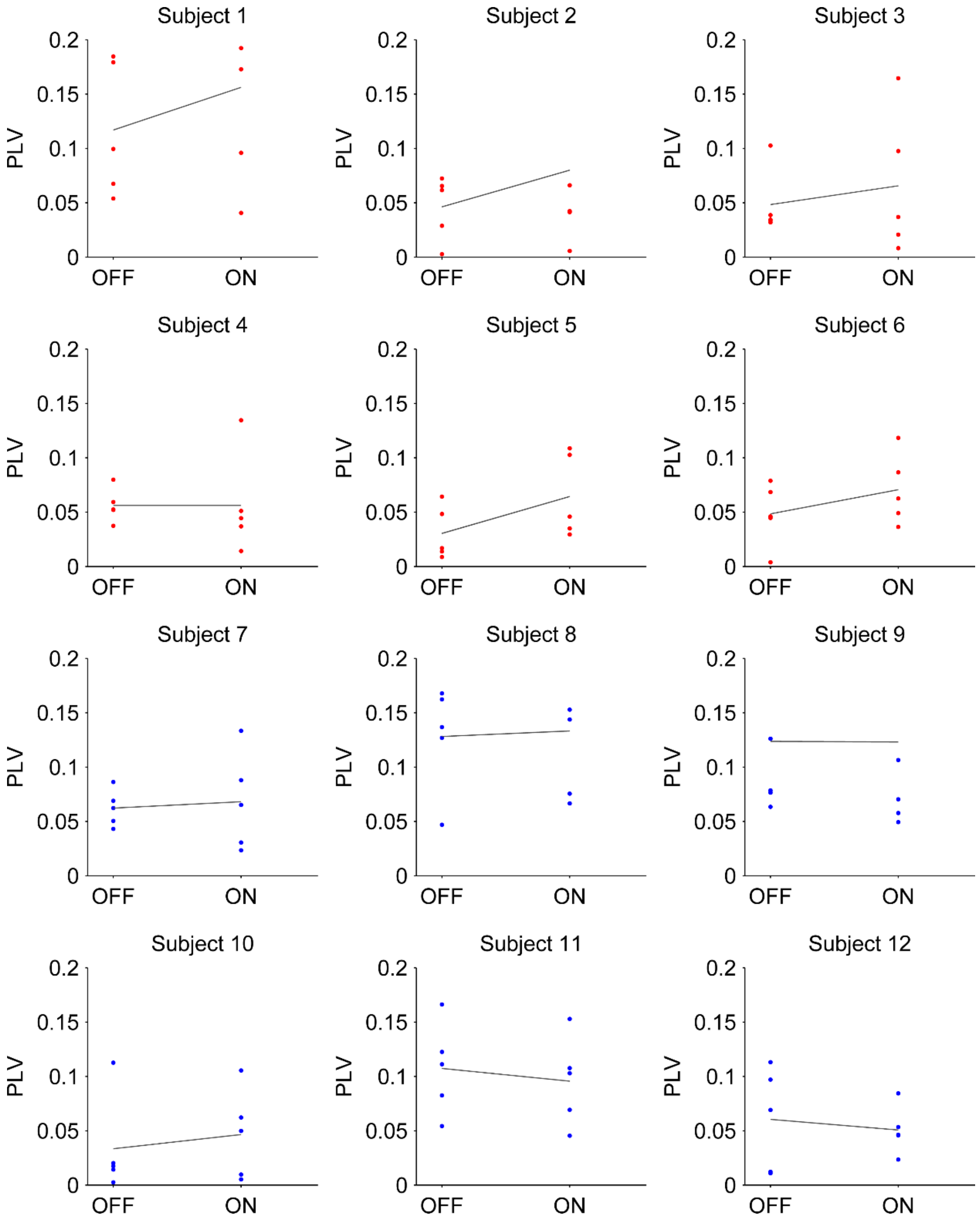

Supplementary Figure 7. Individual subject data showing tACS entrainment of pathological tremor in ET patients: effects of stimulation and topical anesthesia (Experiment 2B). Each panel shows data from one subject. Data from the no anesthesia condition are shown in red (Subjects 1 to 6) and data from the anesthesia condition are shown in blue (Subjects 7 to 12). Single dots represent the PLV value for one of the five repetitions collected for each condition (some data points have a PLV of above 0.2 and thus they are not shown in the graphs). The lines show the mean PLV for each condition. For no anesthesia, five out of six subjects (Subjects 1, 2, 3, 5 and 6) showed an increase in the PLV when stimulation was applied at 2 mA. However, for anesthesia subjects, most subjects showed little or no increase in PLV. Only one subject showed a clear increase (subject 10) in the anesthesia condition.

Experiment 3

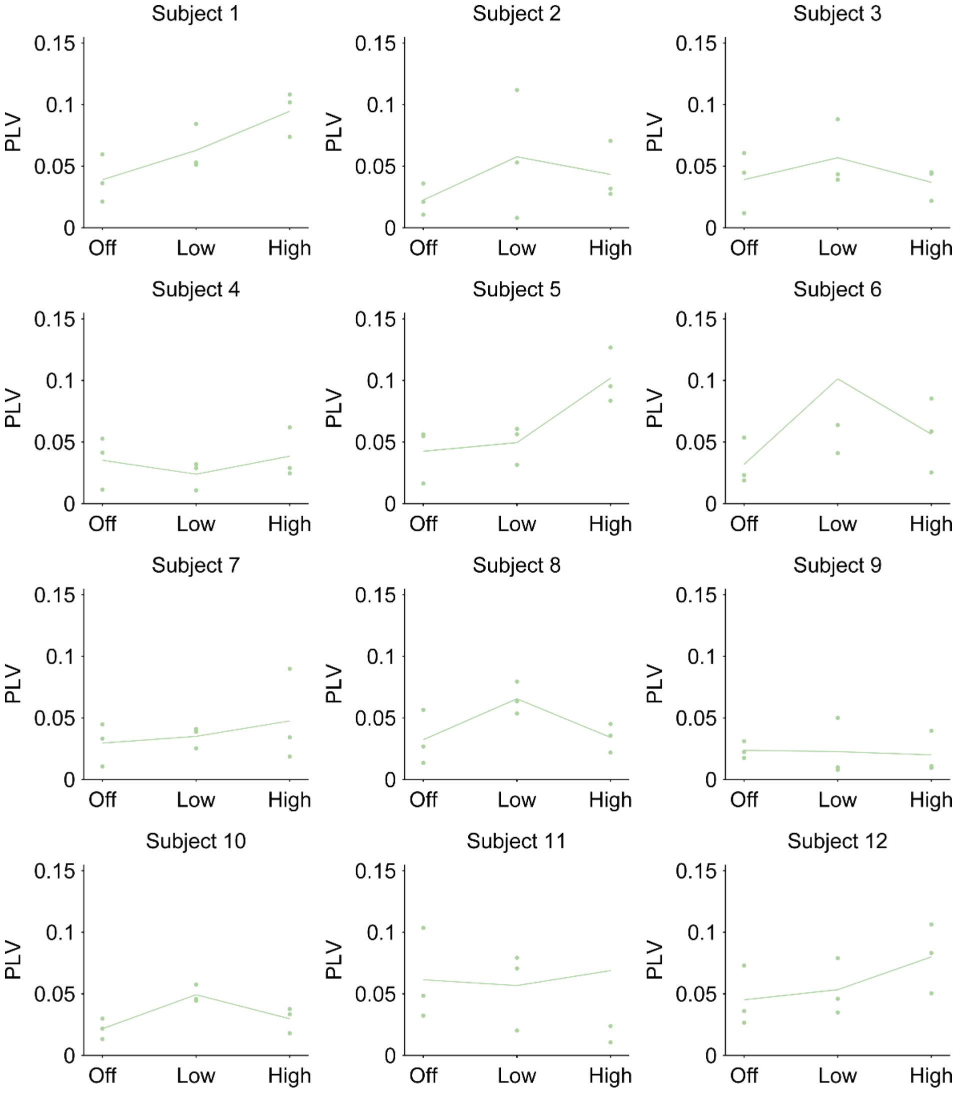

Supplementary Figure 8. Individual subject data showing the effect of transcutaneous-only stimulation on physiological tremor entrainment (Experiment 3). tACS electrodes were placed on the upper arm contralateral to the hand on which tremor was measured, effectively blocking any contribution from a potential transcranial mechanism. Single dots represent the PLV value for one of the three repetitions collected for each amplitude condition (OFF, LOW and HIGH). The lines show the mean PLV for each condition. Similarly to Experiment 2, there is considerable response variability. Some subjects show a large increase in PLV with increasing stimulation amplitude (e.g. Subjects 1, 5 and 6), while others show very little effect (e.g. Subjects 3, 4 and 9). This indicates that even when tACS electrodes are not located on the head we observe very similar patterns of tremor entrainment to those observed with standard tACS. Statistics at the group level (see Results in main text) confirmed these observations. Subject numbering does not correspond to that in Supplementary Figure 3. Four subjects did complete Experiments 2 and 3. These subjects are discussed in the Supplementary Figure 6.

In general, for Experiments 2 and 3 we found that the change in PLV produced by tACS was numerically small, especially if we consider that PLV can range between 0 and 1. However, based on Cohen's d metric we found that the effect sizes of tACS on tremor PLV was in the range of 0.8 to 1.69. This is typically considered a statistically large to very large effect. Thus, while the numerical change in PLV due to tACS are comparatively small, we do measure statistically large and significant effects of tACS on tremor. Additionally, the modulation of this effect with anesthesia is statistically large.

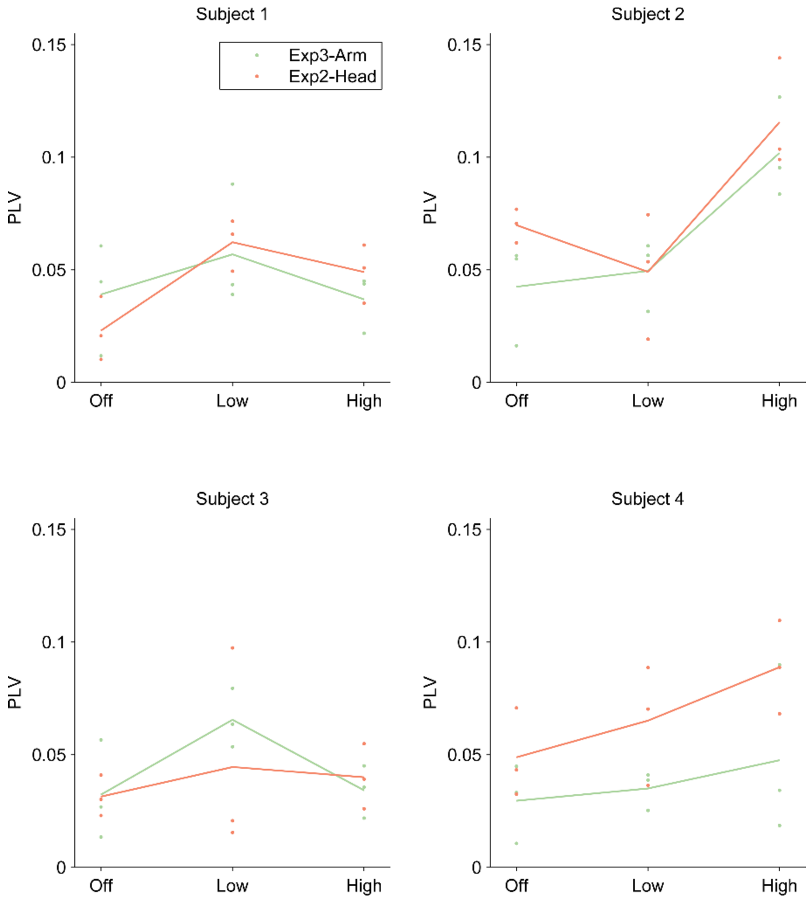

Supplementary Figure 9. Individual data from four subjects who completed both Experiment 2 and Experiment 3. Data from the three amplitude conditions on the no anesthesia day from Experiment 2 are shown in red. Data from the corresponding amplitude conditions in in Experiment 3 are shown in green. Dots show the PLV for each of the three repetitions for each condition and the lines show the mean. Note that as amplitude is increased, the subjects show qualitatively different patterns in their PLV response. Subjects 1 and 3 show the highest PLV for the LOW amplitude conditions. Subject 4 shows a linear increase in PLV as amplitude is increased, while Subject 2 does not show much increase in PLV from the OFF to the LOW condition. What is most striking about the data is that while response patterns clearly differ between individual subjects, the same pattern is observed in Experiments 2 and 3 for each subject. This indicates the location of the tACS electrodes (either on the head in Experiment 2 or on the arm in Experiment 3) has very little effect on the tremor entrainment response. If tACS effects were caused by transcranial stimulation of cortical neurons we would not expect to see this correspondence in data from Experiments 2 and 3. However, if tACS effects were caused by transcutaneous stimulation of peripheral nerves in the skin we could expect to similar response patterns in Experiments 2 and 3. Subject 1 is S1 in Exp2 and S3 in Exp3. Subject 2 is S5 in Exp2 and S5 in Exp3. Subject 3 is S6 in Exp2 and S8 in Exp3. Subject 4 is S7 in Exp2 and S7 in Exp3.

Supplementary Table 6. The amplitudes used to test each of the 12 subjects in Experiment 3 for the low and high amplitude conditions. The last line shows the mean amplitude for each condition tested.

**Subject**	**Low (mA)**	**High (mA)**
1	0.5	2.5
2	0.5	2.5
3	0.5	2.5
4	0.5	2.5
5	0.5	2.5
6	0.5	2.5
7	0.5	2.5
8	0.5	2.5
9	0.5	2.5
10	0.5	2.5
11	0.4	2.2
12	0.5	2.5
**Mean**	**0.49**	**2.47**

Experiment 4

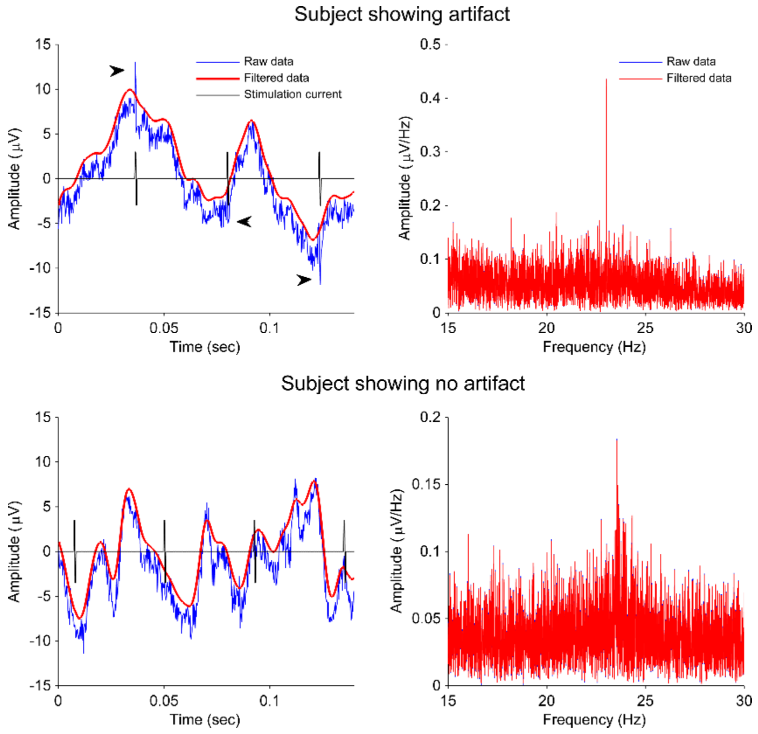

Supplementary Figure 10. The effect of the artifact removal on the EEG signal and its corresponding amplitude Fourier spectrum. The upper left panel shows an example of the raw EEG data (blue line) from a subject who showed a stimulation artifact (black arrows). After low-pass (100 Hz) filtering (red line) the stimulation artifact is completely removed but low-frequency EEG signal of interest is unaffected. The upper right panel shows the Fourier amplitude spectrum for the full of the 3 minute EEG recording in the same subject. A clear peak is visible at the stimulation frequency indicating a neural oscillation at this frequency. The filtered data is shown as red and the raw data is shown as blue However, the two signals overlap completely (i.e. the blue line is not visible) because the low-pass filter does not affect the frequency range of the neural response. The second row shows the same information but for a subject with no visible stimulation artifact in the EEG signal .

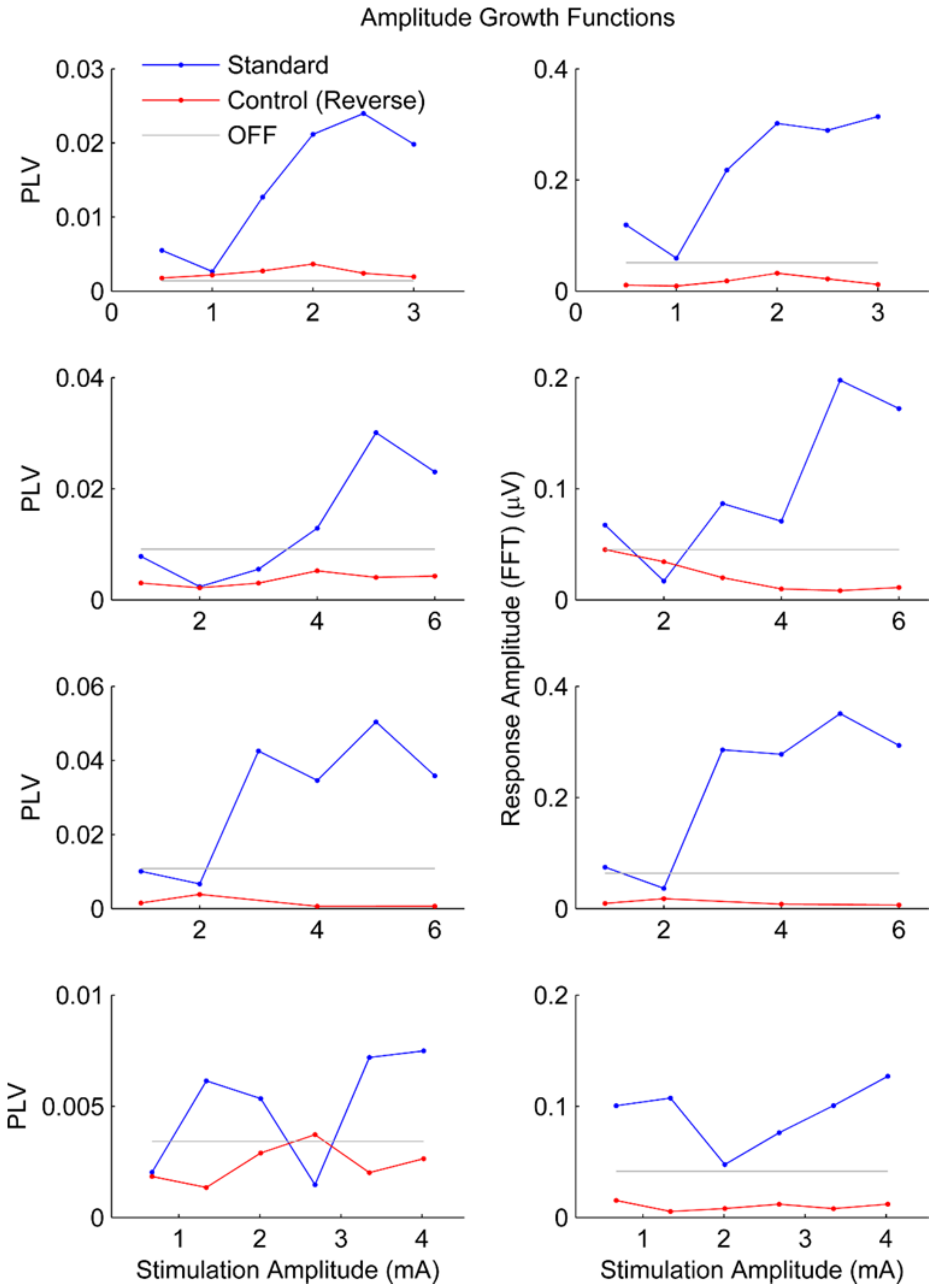

Supplementary Figure 11. To ensure that the results from Experiment 4 were not contaminated by stimulation artifact we performed two different controls in 4 of the subjects.

The first control was the collection of an amplitude growth function. We increased the amplitude of the pulse delivered to the arm over six evenly space amplitude steps from low (either 0.5 or 1 mA) to high (between 3 and 6 mA) and recorded the neural response over the motor cortex (as in Methods, Experiment 4). If we were recording a true neural response, we would expect some subthreshold stimulation amplitudes to show no neural response, and then higher stimulation amplitudes to show a larger neural response. We may also expect to see a non-linear amplitude growth function. However, if our recordings were dominated by artifact we would expect to see some response even for subthreshold amplitudes. We would also expect to see a liner amplitude growth function, since the artifact amplitude will scale linearly as the stimulation amplitude is increased. The results from four subjects are shown as the blue curves. The left panels show the PLV amplitude growth function, the

right panels show the amplitude of the FFT component of the neural response at the stimulation frequency. We found that both metrics show very similar nonlinear amplitude growth functions. We also see that at low stimulation amplitudes the response is not larger that that obtained during the OFF condition (grey line). This indicates that these are subthreshold amplitudes that do not produce a neural response. Thus, the amplitude growth functions indicate that we are recording a true neural response that is not contamined by stimulus artifact.

The second control was to keep all recording and stimulating electrodes in the same position, but to switch the connections so that the electrodes on the arm were now connected to the EEG amplifier and the electrodes on the scalp were connected to the stimulator. With this reversed configuration, because of the principals of electrical reciprocity, any stimulation artifact caused by volume conduction should be exactly the same as with the standard configuration. However, the neural response from the motor cortex should now be completely absent. Thus, if our response metric were dominated by artifact we should still measure a (false) response with the reversed configuration. However, if there is not artifact present we should not measure a neural response. The red curves show the results from the same three subjects and same two response metrics (PLV and FFT component at the stimulus frequncy) across a range of stimulation amplitudes. It is clear that with this reversed configuration we do not measure any significant (false) neural responses. Note, that the subject shown in the bottom row has very small neural responses and an abnormal amplitude growth function. However, except for one amplitude all the reverse configuration is always lower than the standard configuration. Thus, this second control confirms that there is little if any stimulation artifact present in our neural recordings.

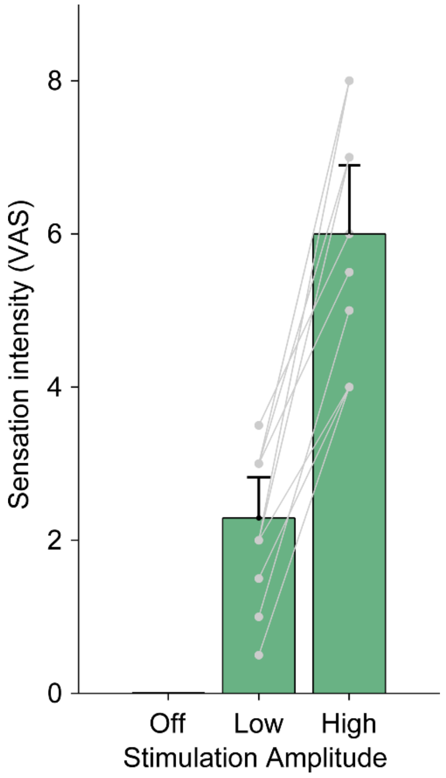

Supplementary Figure 12. Sensation intensity ratings for the single-pulse transcutaneous stimulation. The bar graph shows the mean sensation intensity rating measured using VAS for the single-pulse transcutaneous stimulation across the three conditions (OFF 0; LOW 2.29 ± 0.532; HIGH 6.00 ± 0.89). Dots joined by a line indicate individual subject data. The error bars show confidence intervals.

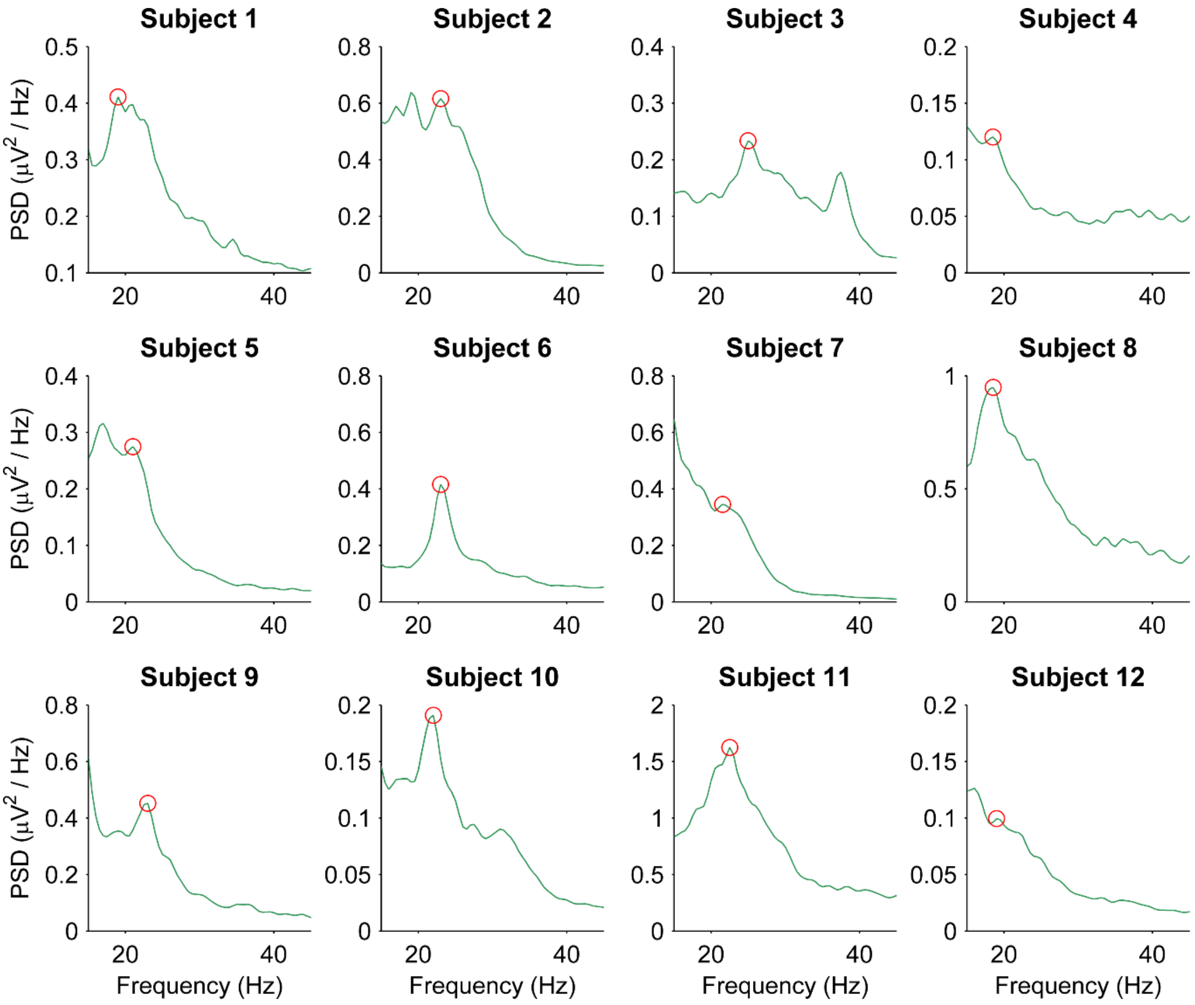

Supplementary Figure 13. Power spectral density (PSD) plots showing the EEG beta band for each individual subject. The PSD (green line) was calculated by recording 3 minutes of EEG without stimulation while the subject was resting. The power spectral density was calculated for 1 second epochs and then averaged over the entire 3 minute recording. Most subjects showed a peak in the beta band frequency range. The red circle indicates the frequency at which rhythmic single-pulse transcutaneous stimulation was delivered.

Supplementary Discussion

Implications for tDCS

Our study investigated the mechanism underlying tACS. Transcranial direct current stimulation (tDCS) is a related neuromodulation method; but instead of alternating current, direct current is passed through the scalp to create a weak, constant, current in the brain. With tDCS the assumed mechanism of action begins with constant polarization of the membrane potential of neurons in the central nervous system (which can then lead to other more long lasting and complex effects). However, peripheral nerves located in the scalp experience much stronger membrane polarization. Most subjects report feeling a tingling sensation when tDCS is switched on[1,2], indicating that this polarization is strong enough to cause action potentials in peripheral nerves. It would be interesting to test if transcutaneous stimulation contributes to any of the reported tDCS effects. Importantly, some tDCS effects are polarity specific: for example, anodic tDCS increases TMS motor evoked potential amplitudes while cathodic tDCS reduces them[3]. An effect mediated by constant polarization of peripheral nerves would be unlikely to show this kind of polarity specific behavior. Thus, the tDCS mechanism of action appears to be different to the tACS mechanism, indicating that tDCS effects are unlikely to be caused by peripheral nerve stimulation.

Improving tACS

Improving our understanding of the tACS mechanism will lead to an improvement in the tACS method. The tACS method currently suffers from a number of limitations, two of which are inter-subject variability and study reproducibility. Within one study there is often a wide range of tACS effect size, with some subjects not showing any effect. In our study 9 of the 12 subjects showed an effect in Experiment 2A. Many tACS studies have also been difficult to reproduce[4] or could not be reproduced[5]. Our results indicate that the transcutaneous mechanism appears to play an important role in causing tACS motor system effects. This improved understanding of the mechanism can now be leveraged to improve the method. For example, it may not be necessary to locate electrodes on the head, nor focus stimulation to a particular brain area. Our results suggest that positioning the electrodes to target one particular peripheral nerve may give stronger, more reproducible effects. Stronger tACS effects, combined with a clearer understanding of the underlying mechanism, should reduce inter-subject variability and increase study reproducibility.

Limitations

Given the differences in skull and skin thickness between rats and humans it is difficult to determine stimulation amplitudes that will produce the same electric field strength in either the brain or the skin. Additionally, the rat model measured tACS effects when the rat was under general anesthesia while our human subjects were fully awake. Rather than trying to exactly match the electric field strengths between the rats and humans, we simply used the rat model to establish that transcutaneous-only stimulation can produce neural entrainment patterns that are similar to those produced by transcranial-only stimulation, albeit under different amplitude conditions than used in human tACS. We then showed that transcutaneous stimulation in humans can entrain tremor and, in a separate experiment, EEG activity. However, we did not prove that transcutaneous entrainment of tremor is caused by peripheral nerves entraining motor cortex activity which then leads to tremor entrainment. It may be that it does; but on the other hand, it may equally well be that it does not and that tremor entrainment in humans occurs via a completely peripheral route not involving the brain. Either way, this limitation does not alter the conclusion that tACS causes tremor entrainment through transcutaneous and not transcranial stimulation.

References

1. Matsumoto, H. & Ugawa, Y. Adverse events of tDCS and tACS: A review. *Clin. Neurophysiol. Pract.* **2,** 19–25 (2017).

2. Brunoni, A. R. *et al.* A systematic review on reporting and assessment of adverse effects associated with transcranial direct current stimulation. *Int. J. Neuropsychopharmacol.* **14,** 1133–1145 (2011).

3. Nitsche, M. A. & Paulus, W. Excitability changes induced in the human motor cortex by weak transcranial direct current stimulation. *J. Physiol.* **527,** 633–639 (2000).

4. Bikson, M. *et al.* Rigor and reproducibility in research with transcranial electrical stimulation: An NIMH-sponsored workshop. *Brain Stimul.* (2017). doi:10.1016/j.brs.2017.12.008

5. Lafon, B. *et al.* Low frequency transcranial electrical stimulation does not entrain sleep rhythms measured by human intracranial recordings. *Nat. Commun.* **8,** 1–13 (2017).
